# Autonomic reactions and peri-interventional alterations in body weight as potential supplementary outcome parameters for thromboembolic stroke in rats

**DOI:** 10.1186/2040-7378-4-7

**Published:** 2012-07-13

**Authors:** Dominik Michalski, Christopher Weise, Carsten Hobohm, Lea Küppers-Tiedt, Johann Pelz, Dietmar Schneider, Johannes Kacza, Wolfgang Härtig

**Affiliations:** 1 Department of Neurology, University of Leipzig, Liebigstr. 20, 04103, Leipzig, Germany; 2 Paul Flechsig Institute for Brain Research, University of Leipzig, Jahnallee 59, 04109, Leipzig, Germany; 3 Institute of Anatomy, Histology and Embryology, Faculty of Veterinary Medicine, University of Leipzig, An den Tierkliniken 43, 04103, Leipzig, Germany

**Keywords:** Thromboembolic stroke, Autonomic changes, Heart rate, Mean arterial pressure, Body weight

## Abstract

**Background:**

Since several neuroprotectives failed to reproduce promising preclinical results under clinical conditions, efforts emerged to implement clinically relevant endpoints in animal stroke studies. Thereby, insufficient attention was given on autonomic reactions due to experimental stroke, although clinical trials reported on high functional and prognostic impact. This study focused on autonomic consequences and body weight changes in a translational relevant stroke model and investigated interrelations to different outcome measurements.

**Methods:**

Forty-eight rats underwent thromboembolic middle cerebral artery occlusion (MCAO) while recording heart rate (HR) and mean arterial pressure (MAP). After assessing early functional impairment (Menzies score), animals were assigned to control procedure or potentially neuroprotective treatment with normobaric (NBO) or hyperbaric oxygen (HBO). Four or 24 hours after ischemia onset, functional impairment was re-assessed and FITC-albumin administered intravenously obtaining leakage-related blood–brain barrier (BBB) impairment. Body weight was documented prior to MCAO and 4 or 24 hours after ischemia onset.

**Results:**

During MCAO, HR was found to increase significantly while MAP decreased. The amount of changes in HR was positively correlated with early functional impairment (*P* = 0.001): Severely affected animals provided an increase of 15.2 compared to 0.8 beats/minute in rats with low impairment (*P* = 0.048). Regarding body weight, a decrease of 9.4% within 24 hours after MCAO occurred, but treatment-specific alterations showed no significant correlations with respective functional or BBB impairment.

**Conclusions:**

Future studies should routinely include autonomic parameters to allow inter-group comparisons and better understanding of autonomic reactions due to experimental stroke. Prospectively, autonomic consequences might represent a useful outcome parameter enhancing the methodological spectrum of preclinical stroke studies.

## **Background**

Stroke is an ongoing health issue with high socioeconomic relevance [1], contributing to 10-12% of deaths in Western countries [2] and accounting for approximately 4% of health-care costs [3]. In focal cerebral ischemia, only tissue plasminogen activator as recanalizing strategy has shown beneficial effects in pharmacological clinical trials (e.g., [4]). On the contrary, numerous potentially neuroprotective drugs have failed to reproduce their impressive preclinical results in randomized clinical trials [5-7], e.g., NXY-059 [8]. This situation might be caused by the complex pathophysiology of acute focal cerebral ischemia with deleterious and beneficial processes occurring simultaneously [5,9], impeding the identification of an exclusively beneficial acting drug. Exploring these complex mechanisms, preclinical studies focused on several ischemia-related key features such as neuronal death [9,10], the integrity of the neurovascular unit [11,12] and diverse aspects of the inflammatory response [9,13,14] – potentially influencing the long-term outcome.

Only little attention was given to autonomic consequences in experimental stroke studies as for example changes in heart rate, arterial blood pressure and temperature. Conversely, Dirnagl [15] and Fisher et al. [6] recommended measurement and control of such parameters throughout preclinical studies, thus enabling comparative analyses of different interventional groups. On the other hand, an alteration of sympathetic/parasympathetic functions in terms of an autonomic imbalance due to acute focal cerebral ischemia, influencing the cardiovascular system and thus the clinical outcome, has been suggested by several clinical studies [16-20]. Particularly, the insula of the right hemisphere was found to affect the autonomic balance in human stroke, as shown by a decreased heart rate variability [16,18,20] and increased rate of cardiac arrhythmias [21-24]. Emphasizing the causal link between arrhythmias and cerebral ischemia, Pasquini et al. [25] eletrocardiographically investigated stroke patients at hospital admission and demonstrated that arrhythmias are more likely a consequence of insular infarctions instead of their underlying cause. Taking these data into account, an improved understanding of ischemia-related alterations of the autonomic system might contribute to an enhanced preclinical stroke research, predominantly addressing pathophysiological issues.

Beyond this perspective, autonomic parameters might also play a role as additional outcome variables in preclinical studies in accordance with the current efforts of using multiple endpoints in order to substantially enhance the study quality [6]. The issue of an extended outcome perspective becomes increasingly important since clinical stroke trials mainly used functional parameters as primary endpoints, in contrast to infarct volume being the central – and in parts exclusive – focus of attention in most preclinical studies [5,6,26]. As a consequence, recent preclinical stroke studies more commonly applied functional tests in order to assess plain neurological deficits (e.g., by Menzies score [27]), or complex behavioral aspects (e.g., by corner test [28]). Strengths and weaknesses of available measurements were extensively discussed in several reviews [29-35]. Searching for further applicable endpoints in preclinical research, the change in body weight as surrogate for general health might play an important role, because this parameter is already in use for detecting critical health conditions [36]. Furthermore, first attempts were made to correlate body weight changes with the routinely used infarct volume [37,38] and functional impairment [38]. Concerning further translational issues in stroke research, the underlying rodent models of focal cerebral ischemia have often been discussed critically: Most previous studies applied techniques with artificial vessel occlusion by electrocautherization or filament insertion, while thromboembolic animal models largely mimic human pathophysiology [26,39,40].

The present study investigated autonomic reactions – in particular changes of heart rate and mean arterial pressure – during right-sided thromboembolic focal cerebral ischemia in rats to explore their natural course and the interrelations with early functional impairment, assessing the significance as potential outcome parameters. Moreover, body weight changes – also representing a promising surrogate for general health following focal cerebral ischemia – were addressed in animals undergoing a potentially neuroprotective treatment with normobaric (NBO) and hyperbaric oxygen (HBO). These interventions were chosen since our previous work [41] has demonstrated differentiated effects on functional impairment and blood–brain barrier (BBB) damage.

## **Methods**

### **Study design and content**

Data from 48 male Wistar rats weighing 307 ± 35 g – taken from our previous work [41] – were retrospectively investigated. The underlying study had been conducted according to the European Communities Council Directive (86/609/EEC) and was approved by local authorities (Regierungspräsidium, Leipzig, Germany). All animals were delivered by Charles River (Sulzfeld, Germany) and subjected to right-sided focal cerebral ischemia by middle cerebral artery occlusion (MCAO) using a thromboembolic model originally described by Zhang et al*.*[42], and modified as previously characterized [43]. Thereby, a polyethylene (PE) tubing with tapered end was advanced via the external carotid artery into the internal carotid artery (ICA) until the origin of the middle cerebral artery. In this position, a weight-adapted blood clot with a mean length of 45 ± 3 mm was injected. Finally, the catheter was removed to restore blood blow in the ICA. During surgery, anesthesia was arranged by using isoflurane (70% N_2_O / 30% O_2_), and temperature maintenance (target temperature 37°C) was ensured with a thermostatically controlled heating pad (Fine Science Tools, Heidelberg, Germany) including a rectal probe. As the first step of the surgical procedure, PE tubes were inserted into the femoral artery for measurement of heart rate and mean arterial pressure, and the femoral vein for future substance application.

**Figure 1:**
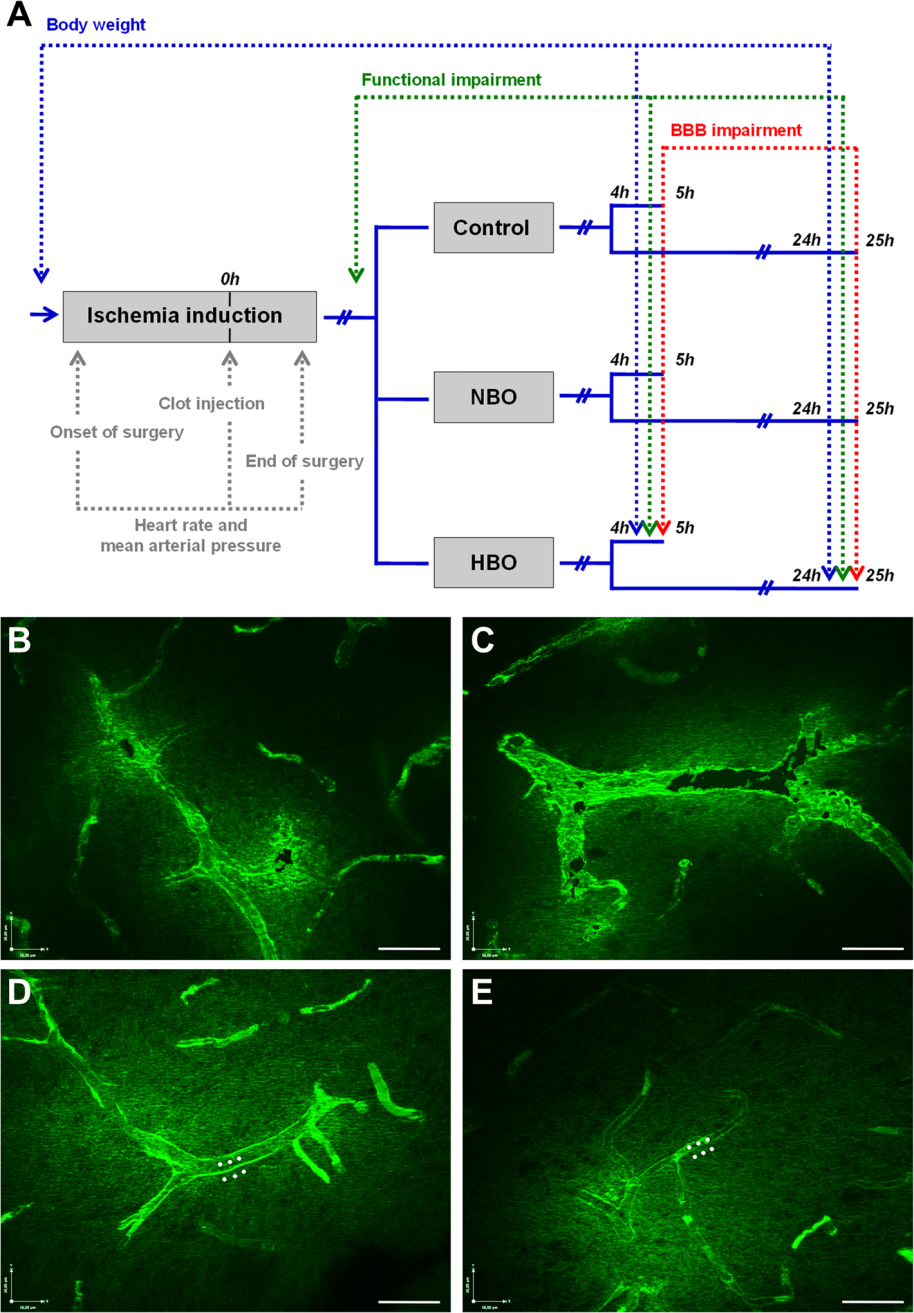
**Study overview (A) with defined time points for assessing heart rate and mean arterial blood pressure, as well as body weight, functional impairment and blood–brain barrier impairment.** Native representative examples **(B, C)** of blood–brain barrier impairment located in the rat brain affected by thromboembolic stroke and clearly indicated by leakage of FITC-albumin from the intra- to the extravasal space at 40x magnification. Further affected vessels **(D, E)** with exemplarily inserted regions of interest for quantification of BBB impairment. Long-term stabilization of the green fluorescent signal of FITC-albumin was ensured by an enhancing technique using carbocyanine 2-anti-FITC IgG. Figures **B-D** were slightly modified by enhancing the green canal on the tone curve using Corel Photo-Paint (Version 12.0, Corel Corp., Ottawa, Canada), applying the same setup for all 4 Figures. Scale bars in B-E: 30 μm.

After MCAO induction and recovery from anesthesia, animals were assigned to control procedure, or treatment with NBO or HBO (n = 16 each). NBO and HBO were started 2 hours after ischemia onset and were performed in a pure oxygen-filled hyperbaric chamber (Sayers/Hebold, Cuxhaven, Germany). NBO was conducted under normal ambient pressure (1.0 absolute atmospheres) and HBO at 2.4 absolute atmospheres, each for 60 minutes. Four hours (n = 8 per treatment group) or 24 hours (n = 8 per treatment group) after ischemia onset, Fluorescein isothiocyanate (FITC)-albumin with a dosage of 20 mg/1 mL saline (Sigma, Taufkirchen, Germany) was administered intravenously for the characterization of BBB impairment. Following a circulation time of usually 1 hour, the animals were sacrificed in deep narcosis. After transcardial perfusion with saline and 4% paraformaldehyde (PFA) in 0.1 M phosphate-buffered saline, pH 7.4 (PBS), the brains were removed from the skull and immersed in PFA overnight, followed by the equilibration in 30% phosphate-buffered sucrose. The complete study setup including procedures and endpoints is summarized in Figure 1A.

### **Study endpoints**

*Heart rate and mean arterial bloodpressure* were assessed during MCAO-related surgery by using a commercial monitoring system (Datex, Helsinki, Finland) connected with the PE tube inserted in the femoral artery. Values were obtained continuously while the following 3 time points were recorded for further calculations: directly after catheter placement (onset of surgery), shortly after MCAO induction (clot injection) and shortly before the surgical procedure was completed (end of surgery).

*Body weight* was measured with a scale (Mettler Toledo BBA425-3SM, Gießen, Germany) prior to surgery (baseline), as well as 4 and/or 24 hours after ischemia induction.

*Functional impairment* was assessed after recovery from surgery-related anesthesia (early functional impairment, M0), as well as 4 hours (M4h) and/or 24 hours (M24h) after ischemia induction by using the Menzies score [27], which is scaled from 0 “no apparent deficits” up to 4 “spontaneous contralateral circling”.

*BBB impairment* was characterized by using FITC-albumin as permeability marker, as previously described in detail [44]. Briefly, serial brain sections of 30 μm thickness were cut coronally using a freezing microtome and stored in 0.1 mol/L Tris-buffered saline, pH 7.4 (TBS) containing sodium azide. To identify sections with clearest infarct presentation as indicated by FITC-albumin leakage, a first screening was performed with a conventional fluorescence microscope (Axioplan, Zeiss, Jena, Germany). For this purpose, sections were extensively washed with TBS, briefly rinsed with distilled water, mounted on fluorescence-free slides, air-dried and coverslipped with Entellan in toluene (Merck, Darmstadt, Germany). Afterwards, the neighboring serial sections were washed with TBS, blocked with TBS containing 2% bovine serum albumin and 0.3% Triton X-100 (TBS-BSA-T) for 1 hour. Subsequently, the green fluorescent signal was enhanced by incubation with carbocyanine (Cy)2-anti-FITC IgG (20 μg/mL TBS-BSA-T; Jackson ImmunoResearch, West Grove, PA) for 1 hour. The sections then were washed, mounted and coverslipped as described above. The ratio of fluorescence-based paravasal and intravasal intensity of enhanced FITC-albumin served as surrogate for BBB impairment, analyzed by a fluorescence microscope (Axioplan 2, Zeiss) including structured-light confocal system (OptiGrid, Qioptiq, Fairport, NY), digital camera (ORCA-ER, Hamamatsu, Hamamatsu City, Japan) and imaging software (Volocity 4.3, PerkinElmer, Waltham, MA). In detail, routinely 3 vessels with clearest FITC-albumin leakage in the lesion-bordering zone were selected in each animal. For each of these vessels, measurements were done with 3 cylindrical regions of interest (ROIs; diameter 2 μm, 9 confocal layers, z-interval 0.5 μm) that were positioned in the paravasal space with a distance of approximately 2 μm from the vessel wall, and 3 ROIs that were positioned directly in the vessel (Figure 1B-E). Finally, the assessed mean intensities within the ROIs (setup: 40x magnification, further digital zoom if required, water immersion objective with numerical aperture 1.4, exposure time 80 ms, gain 0, offset 0, super grid quality, grid gain 3x, excitation Cy2 480 nm, emission 527 nm, excitation Cy3 545 nm, emission 610 nm) were used to calculate means which served as source for the extra-/intravasal ratio.

### **Statistical analysis**

Calculations were made with SPSS (Version 18.0, SPSS Inc., IBM Company, Chicago, IL). Wilcoxon test, Mann–Whitney *U* test and one-way analysis of variance (ANOVA) were performed for intra- and inter-group differences. Pearson correlations and partial correlations were used for testing interrelations. Overall, a *P* < 0.05 was considered as statistically significant. Data are given as mean ± standard deviation, unless otherwise indicated.

## **Results**

The surgical procedure for inducing right-sided thromboembolic stroke was performed with a mean duration of 69 ± 22 minutes. In parallel, the body temperature was kept stable from onset (37.2 ± 0.4°C) to the end of surgery (37.4 ± 0.3°C), although this marginal increase achieved statistical significance (Wilcoxon test, *P* = 0.003).

**Figure 2:**
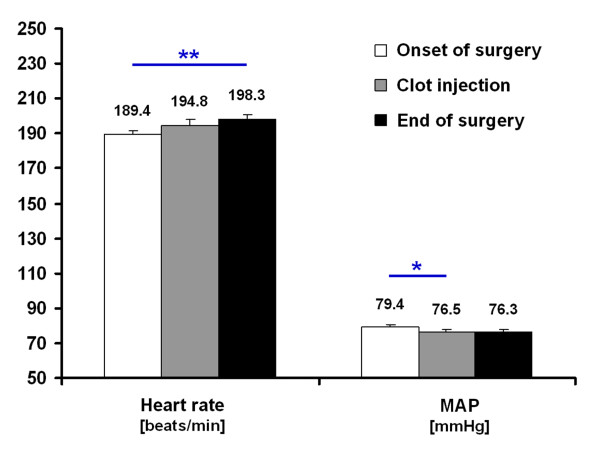
**Course of heart rate and mean arterial pressure (MAP) during surgery inducing right-sided thromboembolic stroke**Bars represent means and added lines indicate standard errors. Sample sizes (time point: heart rate / MAP): onset of surgery: n = 47 / n = 47; clot injection: n = 45 / n = 45; end of surgery: n = 44 / n = 44. ^*^P < 0.05; **P < 0.01.

**Figure 3:**
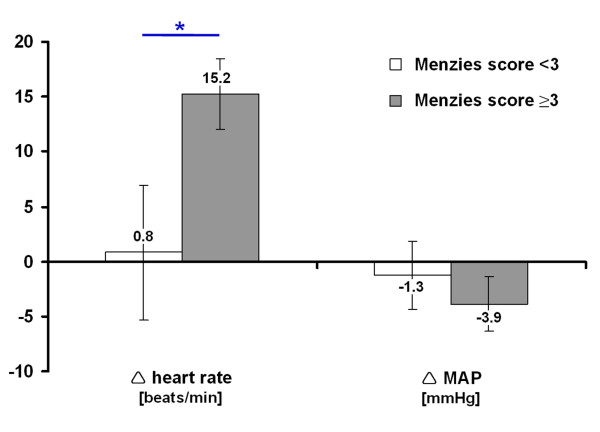
**Intra-surgical changes in heart rate and mean arterial pressure (MAP) depending on early functional outcome, divided into a group with low deficit (Menzies score <3) and severe deficit (Menzies score ≥3).** Bars represent means and added lines indicate standard errors. Sample sizes (group: heart rate / MAP): Menzies score <3: n = 17 / n = 17; Menzies score ≥3: n = 27 / n = 27.^*^P < 0.05.

Obtained changes of heart rate and mean arterial pressure during ischemia induction are shown in Figure 2. In the overall sample, a significant increase in heart rate of about 9 ± 21 beats/minute occurred from onset to the end of surgery (Wilcoxon test, *P* = 0.006). In contrast, the mean arterial pressure declined about 3 ± 12 mmHg during surgery with a significant change between onset of surgery and clot injection (Wilcoxon test, *P* = 0.039). Interestingly, the change in heart rate was clearly correlated with early functional impairment following right-sided thromboembolic stroke (M0; Pearson, r = 0.50; *P* = 0.001) representing an explained variance of 25%, while the change in mean arterial pressure did not show a significant correlation (Pearson, r = 0.06; *P* = 0.684). Figure 3 displays this relationship in a more detailed manner while separating the early functional impairment into a group with low deficit (Menzies score <3) and severe deficit (Menzies score ≥3). Here, the observed increase in heart rate in subjects with severe early functional impairment became even clearer (Mann–Whitney *U* test, *P* = 0.048). Additionally, the interrelations between changes in heart rate and mean arterial pressure, as well as their relationship to FITC-albumin leakage as surrogate for BBB impairment were analyzed in the whole sample. A non-significant correlation was found between changes in heart rate and mean arterial pressure (Pearson, r = 0.15; *P* = 0.336). Furthermore, neither the change of heart rate (Pearson, r = 0.20; *P* = 0.195) nor the change of mean arterial pressure (Pearson, r = −0.07; *P* = 0.646) was significantly correlated with BBB impairment.

**Figure 4:**
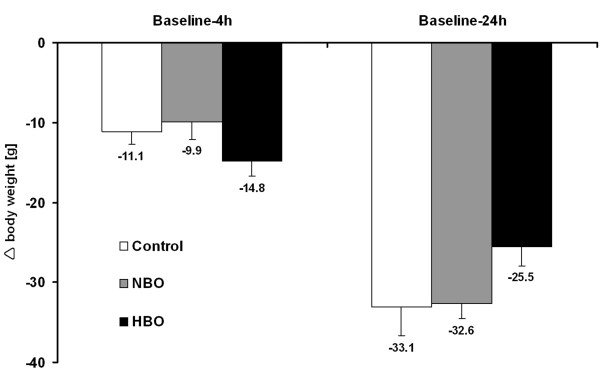
**Changes from baseline body weight to 4 and 24 hours depending on assigned treatment.** Bars represent means and added lines indicate standard errors. Sample sizes (treatment group: baseline-4 h / baseline-24 h): control: n = 8 / n = 8; NBO: n = 8 / n = 8; HBO: n = 8 / n = 8.

**Figure 5:**
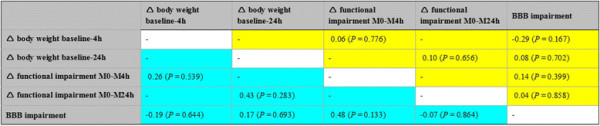
**Correlation matrix between changes (∆) in body weight, functional impairment and blood–brain barrier (BBB) impairment in the overall sample (color-coded in yellow) and the control group (color-coded in turquoise).** Functional impairment was assessed according to Menzies et al. [27]; BBB impairment was measured as previously described by Michalski et al. [41,44]; sample sizes for calculations in the overall sample: ranging from n = 23 to n = 38, and for calculations in controls: ranging from n = 8 to n = 11.

**Figure 6:**
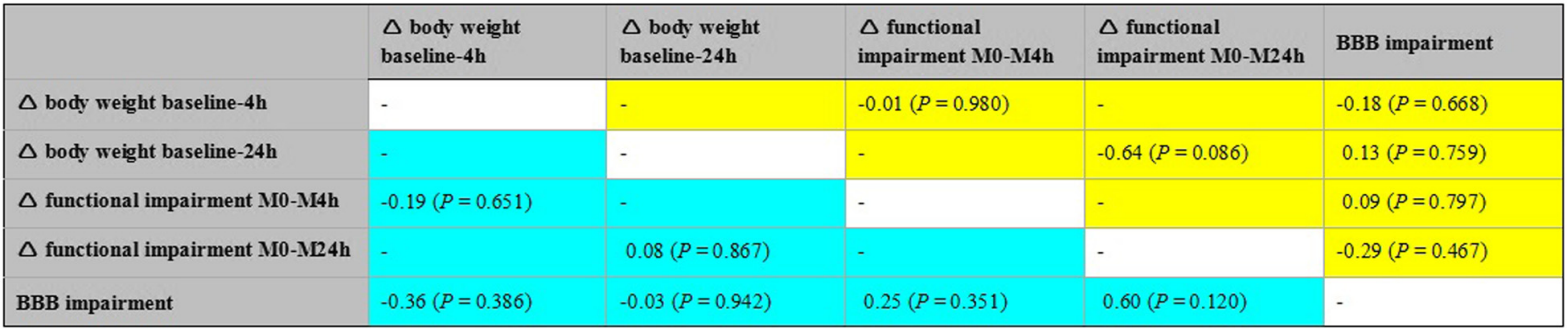
**Correlation matrix between changes (∆) in body weight, functional impairment and blood–brain barrier (BBB) impairment in animals which underwent treatment with NBO (normobaric oxygen; color-coded in yellow) and HBO (hyperbaric oxygen; color-coded in turquoise).** Functional impairment was assessed according to Menzies et al. [27]; BBB impairment was measured as previously described by Michalski et al., [41,44]; sample sizes for calculations in NBO-assigned animals: ranging from n = 8 to n = 11, and for calculations in HBO-assigned animals: ranging from n = 7 to n = 16.

In the overall sample, body weight as surrogate for general health was found to decrease of about 12 ± 6 g from baseline to 4 hours (Wilcoxon test, *P* = 0.000) and about 31 ± 8 g from baseline to 24 hours (Wilcoxon test, *P* = 0.000), which represents a loss of 4.1% and 9.4%, respectively, from baseline body weight. Due to the finding that baseline body weight did not differ between the treatment groups (NBO, HBO) and the control group (ANOVA, F = 2.1; *P* = 0.129), the obtained changes were analyzed according to the 3 interventional groups (Figure 4). Thereby, a tendency for decreased loss of body weight during the first 24 hours after ischemia onset was found in the HBO group, although the overall changes were not statistically significant (baseline to 4 hours: ANOVA, F = 1.8; *P* = 0.189; baseline to 24 hours: ANOVA, F = 2.3; *P* = 0.130). Figures 5 and 6 summarize the interrelations between changes in body weight and functional impairment as well as BBB impairment, which were addressed since the time point-corrected correlation of functional impairment at 4/24 hours and BBB impairment at 5/25 hours revealed a significant coefficient (partial correlation, r = 0.37; *P* = 0.011) in the overall sample. Unexpectedly, changes in body weight were not significantly associated with changes in functional impairment or BBB impairment (Figure 5, color-coded in yellow). To consider potential treatment effects, the coefficients were separately calculated for each interventional group. Here, the lack of significant coefficients remained when focusing on control-proceeded (Figure 5, color-coded in turquoise) and NBO- or HBO-treated (Figure 6) animals separately. Therefore, treatment-associated changes in functional or BBB impairment were not sufficiently reflected by simultaneous changes in body weight. The differently diminished impairment within 24 hours after ischemia onset was predicted by 18.5% (explained variance) of change in body weight in control-proceeded subjects, as compared to 41.0% in NBO- and 0.6% in HBO-treated animals. Remarkably, the underlying correlation coefficient in animals receiving NBO was negative (r = −0.64), in contrast to controls which were characterized by a positive coefficient (r = 0.43).

## **Discussion**

The first part of the present study aimed on autonomic consequences – particularly heart rate and mean arterial blood pressure – during right-sided focal cerebral ischemia in rats. A thromboembolic stroke model was applied with the objective to mimic the human pathophysiology as best as possible [26,40]. In this regard, right-hemispheric cerebral ischemia was of special interest since clinical data indicated autonomic reactions predominantly in ischemic stroke involving the right hemisphere when compared to the left [16,18,20,22-24]. Although changes in body temperature appear interesting in this context [37,45,46], this parameter was controlled during the surgical procedure using a heating pad to avoid anesthesia-related cooling, which meets the standard of care in this field [6,15].

The first main finding of the present study was a significant increase in heart rate and decrease in mean arterial pressure during the induction of right-sided thromboembolic stroke in rats representing most probably specific ischemia-related autonomic alterations, which have – to the authors’ best knowledge – not been reported previously for this model. These results provide evidence for such reactions in the applied stroke model, which appears relevant for translational issues since clinical studies also described autonomic imbalances after right-hemispheric ischemia, especially involving the insula [16,18,20-24]. Summarizing the few available data from animal research that have addressed autonomic changes after focal cerebral ischemia [47], it is remarkable that probably the used different animal species and strains, time points of assessing arterial pressure and heart rate, sides of infarction, and especially the most commonly used invasive and hence artificial MCAO model led to inconsistent results. In detail, Butcher et al. [48] reported a decrease in mean arterial pressure 5 hours following right-sided MCAO in spontaneously hypertensive rats, which could not be replicated by the authors when using Wistar-Kyoto rats. The same authors also found an increase of heart rate starting 2 hours after MCAO in spontaneously hypertensive rats, but again this effect was not observed in Wistar-Kyoto rats [48]. On the other hand, Hachinski et al. [49] performed right-sided MCAO in rats which led to an increased mean arterial pressure 4 hours after ischemia onset when compared to controls and left-sided ischemia. In a further study by Cechetto et al. [50], mean arterial pressure did not significantly differ from −30 to 90 and 180 minutes following left-sided MCAO only, but a decreasing course was observed following sham procedure or occlusion of the ipsilateral common carotid artery. With respect to the clinically demonstrated relevance of the insular cortex [19-21,23], Smith et al. [51] performed left-sided MCAO in cats and showed that insular involvement results in increased catecholamine plasma levels when compared to non-insular infarction. Interestingly, the present study revealed a correlation between the amount of changes in heart rate during right-sided thromboembolic stroke and early functional outcome (see Figure 3), indicating a quantitative and not only qualitative relationship. The derived hypothesis of a link between the amount of autonomic reactions and size of affected brain tissue is supported by a recent study from Purins et al. [52]: While standardizing a brain death model in pigs, a growing intracranial pressure due to an increased intracranial volume led to an increase in heart rate as well as a decrease in mean arterial and cerebral perfusion pressure – finally disrupting the cerebral blood flow. Taken together, the first part of our study yields the suggestion to routinely record autonomic parameters in future preclinical stroke studies, first, to report on potential inter-group differences in efficacy-aimed approaches, and second, to explore interrelations of focal cerebral ischemia and autonomic consequences in approaches primarily addressing pathophysiological issues. For the latter one, visualization tools for cerebral vessel occlusions and related blood flow phenomena (e.g., magnetic resonance imaging-based angiography and laser Doppler flowmetry) might be appropriate to establish the causal link. The presented findings further indicate that autonomic reactions in thromboembolic stroke models might be useful as additional outcome parameters, prospectively extending the study spectrum methodologically.

The second part of the present study dealt with changes in body weight, which represents an already used surrogate for general health [36]. Therefore, this parameter might also be a useful outcome measurement in preclinical stroke studies. In the overall sample, a decrease in body weight of about 4% during the first 4 hours, and about 9% during the total 24-hour observation period was found. Comparable results were published by Overgaard et al. [38], who determined a weight loss between 4.8 and 5.9% within 24 hours after thromboembolic right-sided MCAO in rats. Additionally, Lubjuhn et al. [53] assessed body weight changes in mice subjected to distal MCAO and provided a loss of about 6.6% during the first 24 hours after ischemia onset. Furthermore, Dittmar et al. [45] reported markedly higher values of body weight loss (approximately 15–20%) 2 days following MCAO in rats when compared to sham-operated animals (about 5%). However, differing post-surgical changes in body weight have caused an ongoing discussion on underlying mechanisms – amongst others the applied models of ischemia appear to play an important role in this context [32,37,45,54]. Besides the frequently discussed issue of early MCAO-induced weight loss, a study by Boyko et al. [55] also focused on the long-term recovery of body weight and confirmed model-related differences also at day 14 and 28 after ischemia onset. To explore whether early body weight changes − as shown here − are useful to differentiate treatment effects following thromboembolic stroke, the influence of a potentially neuroprotective treatment with NBO and HBO has been investigated. This approach became interesting since the administration of NBO (60 minutes, starting 2 hours after ischemia onset) led to a significantly improved functional impairment during a 24-hour observation period, while controls showed a progressive stroke with significantly worsened impairment and no functional changes were observed in HBO-treated animals [41]. Concerning a further relevant outcome parameter, in the same study NBO and HBO caused a tendency to reduce BBB impairment, as indicated by the extra-/intravasal ratio of FITC-albumin, while this effect was absent in the control group [41]. Unfortunately, the present study revealed no significant differences of ischemia-related body weight changes between controls, NBO- and HBO-treated animals. To further explore this finding, correlations have been calculated between changes in body weight, functional and BBB impairment (Figures 5 and 6) – overall resulting in non-significant interrelations. Taking the obtained tendencies during the 24-hour observation period into account, it is noteworthy that NBO resulted in a negative coefficient (r = −0.64), hence the functional improvement in this group was associated with decreasing body weight loss. In contrast, an inverse situation was observed in the control group (r = 0.43), indicating here that functional worsening was associated with increasing weight loss. In general, the observed lack of significant interrelations between changes in body weight and 2 further clinically relevant parameters limits the usefulness of body weight as sensible outcome parameter. However, this finding needs to be discussed critically, since previous studies have demonstrated significant correlation coefficients of 0.9 [37] and 0.59 [38] between weight loss and infarct volume. These differences might be caused by different species investigated, time points of assessment and ischemia models: mice, 72 hours after transient MCAO [37] vs. rats, 24 hours after thromboembolic MCAO, partially involving the internal carotid artery [38]. Taken together, further research is required to identify specific mechanisms of body weight changes following experimental stroke, and to explore the interactions of these changes and other outcome parameters. This approach should preferably include both short- as well as long-term observations.

The present study has some limitations: The retrospective design might have produced bias and, therefore, the obtained results need to be confirmed in a prospective setting including an extended catalog of autonomic parameters (e.g., breathing frequency, heart rate variability). The use of potentially neuroprotective oxygen at 2 different ambient pressures allows only conclusions with respect to these interventions. However, the application of oxygen at other ambient pressures or the use of alternative neuroprotective approaches might have provided different changes in body weight or substantial interrelations with both functional and BBB impairment.

## **Conclusions**

In summary, this study demonstrated a significant increase in heart rate and decrease in mean arterial pressure during induction of right-sided thromboembolic stroke in rats, indicating specific autonomic alterations in this clinically relevant model. Interestingly, the amount of changes in heart rate was significantly associated with early functional outcome and therefore qualifies as potential outcome parameter in future stroke studies. Concerning translational issues in stroke research, these findings become relevant since autonomic consequences of stroke models often remained unattended in preclinical studies, whereas clinical studies have shown autonomic imbalances due to right-hemispheric cerebral ischemia with prognostic impact. In addition, body weight was found to be decreased due to thromboembolic cerebral ischemia but cannot be considered as sensitive outcome parameter in different oxygen-related treatment approaches.

Further studies are required to investigate autonomic consequences in rodent stroke models in more detail by using extended panels of parameters, and with special emphasis on potentially influencing factors like lesion side, size, vessel status, resulting blood flow alterations and different neuroprotective approaches.

## Competing interests

The authors declare that there are no competing interests.

## Authors’ contributions

DM, CH, JK and WH designed the underlying study, and DS facilitated funding. The study focus of the present retrospective analysis (autonomic and body weight alterations) originated from LKT and DM. DM and CW conducted the underlying animal experiments, and thereby, collected data on body weight, heart rate, arterial pressure and functional outcome. JP and WH performed tissue preparation and serial staining, JP further assessed FITC-albumin extravasation in a blinded manner. DM analyzed the data and wrote the manuscript, followed by a critical revision by WH and CW. All authors read and approved the final manuscript.
